# The Influence of Temperature and Viscosity of Polyethylene Glycol on the Rate of Microwave-Induced In Situ Amorphization of Celecoxib

**DOI:** 10.3390/molecules26010110

**Published:** 2020-12-29

**Authors:** Nele-Johanna Hempel, Tra Dao, Matthias M. Knopp, Ragna Berthelsen, Korbinian Löbmann

**Affiliations:** 1 Department of Pharmacy, University of Copenhagen, 2100 Copenhagen, Denmark; nele.hempel@sund.ku.dk (N.-J.H.); htdao@outlook.de (T.D.); ragna.berthelsen@sund.ku.dk (R.B.); 2 Bioneer:FARMA, Department of Pharmacy, University of Copenhagen, 2100 Copenhagen, Denmark; mmk@bioneer.dk

**Keywords:** in situ amorphization, microwave radiation, polyethylene glycol, viscosity, temperature, dissolution, monotecticum, amorphous solid dispersion

## Abstract

Microwaved-induced in situ amorphization of a drug in a polymer has been suggested to follow a dissolution process, with the drug dissolving into the mobile polymer at temperatures above the glass transition temperature (*T*_g_) of the polymer. Thus, based on the Noyes–Whitney and the Stoke–Einstein equations, the temperature and the viscosity are expected to directly impact the rate and degree of drug amorphization. By investigating two different viscosity grades of polyethylene glycol (PEG), i.e., PEG 3000 and PEG 4000, and controlling the temperature of the microwave oven, it was possible to study the influence of both, temperature and viscosity, on the in situ amorphization of the model drug celecoxib (CCX) during exposure to microwave radiation. In this study, compacts containing 30 wt% CCX, 69 wt% PEG 3000 or PEG 4000 and 1 wt% lubricant (magnesium stearate) were exposed to microwave radiation at (*i*) a target temperature, or (*ii*) a target viscosity. It was found that at the target temperature, compacts containing PEG 3000 displayed a faster rate of amorphization as compared to compacts containing PEG 4000, due to the lower viscosity of PEG 3000 compared to PEG 4000. Furthermore, at the target viscosity, which was achieved by setting different temperatures for compacts containing PEG 3000 and PEG 4000, respectively, the compacts containing PEG 3000 displayed a slower rate of amorphization, due to a lower target temperature, than compacts containing PEG 4000. In conclusion, with lower viscosity of the polymer, at temperatures above its *T*_g_, and with higher temperatures, both increasing the diffusion coefficient of the drug into the polymer, the rate of amorphization was increased allowing a faster in situ amorphization during exposure to microwave radiation. Hereby, the theory that the microwave-induced in situ amorphization process can be described as a dissolution process of the drug into the polymer, at temperatures above the *T*_g_, is further strengthened.

## 1. Introduction

In situ amorphization is an approach to obtain the amorphous form of a drug in the final dosage form. Using this approach, the common stability and manufacturing problems associated with the amorphous form can be circumvented. This includes, but is not limited to, problems concerning the poor flowability of amorphous powders and the stability, either due to a drug loading above the thermodynamic solubility of the drug in the polymer or the hygroscopicity of certain polymers used to stabilize the amorphous form of the drug. In theory, higher drug loadings than the thermodynamic solubility at room temperature can be made accessible for drug administration using the in situ amorphization approach, as only short-term stability is necessary. Presently, in situ amorphization has been demonstrated by immersion in water, by convectional heating and upon exposure to microwave radiation [1,2,3,4].

Microwave-induced in situ amorphization is based on the absorption of microwave radiation by a dielectric excipient [5]. Household microwave ovens, as well as most microwave ovens for organic synthesis (as used in this study), work at a fixed frequency of 2.45 GHz, whilst the microwave radiation covers the range from 0.03 to 300 GHz in the electromagnetic spectrum [6]. In order for a material to respond to microwave radiation, it needs to absorb microwave radiation, i.e., the material needs to exhibit dielectric properties. Such materials usually contain molecules with dipoles, which will adapt to an alternating electromagnetic field, causing frictions of the molecules and subsequently the generation of heat [7,8]. The most common microwave responding dipole is water, which, for example in the form of sorbed water, has been shown to act as a heating source and plasticizer when preparing a fully amorphous solid dispersion (ASD) by exposure of compacts containing celecoxib (CCX) and polyvinylpyrrolidone to microwave radiation for 10 min [2]. Recently, glycerol has also been successfully used as a heating source for microwave-induced in situ amorphization [4]. The absorption of microwave radiation by the drug is considered negligible [9], however, certain polymers, such as the semicrystalline polymer polyethylene glycol (PEG), can absorb microwave radiation to a certain extent, especially at temperatures above the melting point (*T*_m_) of the polymer, as the molecules at these temperatures can more easily adapt to the alternating electromagnetic field [7,10,11].

It has been proposed that microwaved-induced in situ amorphization is governed by the dissolution of the crystalline drug particles (solute) into the polymeric network (solvent) above the glass transition temperature of the polymer (*T*_g_) [2,12,13]. Thus, the rate of amorphization may be described by the Noyes–Whitney equation (Equation (1)) and will be influenced by the surface area of the crystalline drug particles, the diffusion coefficient of the dissolved drug molecules in the polymer, the thickness of the diffusion layer, as well as the solubility of the drug in the polymer and the concentration gradient [14]. In accordance, Hempel et al. (2020) have shown that increasing the surface area of the drug particles increases the rate of in situ amorphization, which is advantageous for obtaining a fully amorphous ASD during the relatively short exposure times applied for in situ amorphization using microwave radiation.

As diffusion of a drug in solution can mathematically be described by the Stokes–Einstein equation (Equation (2)), it is apparent that the rate of amorphization will also depend on (i) the temperature and (ii) the viscosity of the solvent [15], i.e., the polymer in the case of in situ amorphization using microwave radiation and the size of the diffusion molecule, i.e., the drug CCX.

(1) $$\mathrm{Noyes}-\mathrm{Whitney}\;\mathrm{Equation}:\;\frac{dm}{dt}=A\times \frac{D}{h}\times \left(C_{s}-C_{b}\right)$$

(2) $$\mathrm{Stoke}-\mathrm{Einstein}\;\mathrm{Equation}:\;D=\;\frac{k}{6\pi r}\times \frac{T}{\eta }$$

where *dm/dt* = solute dissolution rate, *m* = mass of dissolved material, *t* = time, *A* = surface area of the solute particle, i.e., the crystalline drug particles, *h* = thickness of the diffusion layer, *C_s_* = particle surface (saturation) concentration, *C_b_* = concentration in the bulk solvent/solution; i.e., drug concentration in polymer, *D* = Diffusion coefficient of the solute in solution, *k* = Boltzmann constant, *T* = absolute Temperature, *η* = viscosity of the solvent, *r* = radius of the solute molecule.

In this study, two different molecular weights of the semicrystalline polymer PEG were used with the model drug CCX. Both PEGs have similar melting points, however, different viscosities at the same temperatures in their melt due to their different molecular weights and chain lengths. As the same model drug, CCX, at the same drug load, 30 wt%, was used in combination with the two polymers, PEG 3000 and PEG 4000, and, as the solubility of the drug in this polymer is independent of the molecular weight of the polymer [16], it was assumed that the surface area and the thickness of the diffusion layer were similar in the two polymers. Furthermore, the size of the diffusion molecule, i.e., CCX, is the same. Hence, the rate of amorphization of CCX into either PEG 3000 or PEG 4000, was expected to only be affected by the temperature and the viscosity of the polymer.

To evaluate if the in situ amorphization process of a drug in a polymer in fact is governed by a dissolution process, the purpose of this study was to investigate the influence of the temperature and the viscosity on the in situ amorphization of CCX, using PEG 3000 and PEG 4000. This was accomplished by exposing compacts containing 30 wt% CCX, 69 wt% PEG 3000 or PEG 4000 and 1 wt% lubricant to microwave radiation at different target temperatures, and determine the degree of amorphization.

## 2. Results and Discussions

PEG is a semicrystalline polymer with a *T*_m_ below 70 °C regardless of molecular weight [17]. The onset melting points for PEG 3000 and PEG 4000 were both found to be at around 53 °C (Figure S1), and the onset melting points for 30 wt% CCX with 70 wt% PEG 3000 or PEG 4000 were between 41–44 °C (Figure S2). Recently, the equilibrium solubility of CCX in PEG 300 (liquid at room temperature) was reported to be around 40 wt% [18]. As drug solubility in a polymer was found to be independent of the molecular weight of the polymer, the solubility of CCX in PEG 3000 and PEG 4000 is also assumed to be around 40 wt% [16]. Therefore, a drug load of 30 wt% CCX was chosen for the present study, which is below the equilibrium solubility at room temperature.

Both semicrystalline PEG 3000 and PEG 4000 have a *T*_g_ below 0 °C (e.g., PEG 4000 has a reported *T*_g_ of −76.6 °C [19]) for the small amorphous fraction. This allows a fraction of the drug to dissolve into the small amorphous fraction of the polymer already at room temperature (as this is above the *T*_g_) [3], during compaction (partial melting of the crystalline fraction of PEG can occur due to the increased pressure) [2], and before exposure to microwave radiation. Upon exposure to microwave radiation, the temperature of the compact increased, and the viscosity of the polymers decreased (markedly at temperatures above the *T*_m_ of PEGs), which collectively increased the rate of CCX dissolution/amorphization into the molten PEGs.

### 2.1. The Influence of Viscosity on In Situ Amorphization of CCX in PEG (Target Temperature)

**Figure 1:**
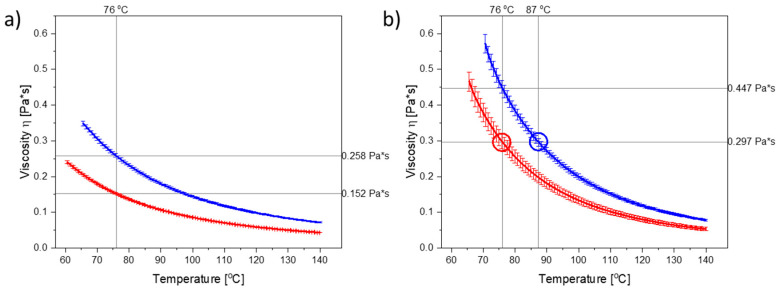
Temperature-dependent viscograms obtained at a shear rate of 100 s^−1^ for (**a**) PEG 3000 (red) and PEG 4000 (blue) and (**b**) PEG 3000 with 30 wt% CCX (red) and PEG 4000 with 30 wt% CCX (blue). Reference lines indicate the temperatures and viscosities used in this study. Mean ± SD (*n* = 3).

The viscosity of PEG is depending on the molecular weight [20]. Figure 1a illustrates the temperature-dependent viscograms for molten PEG 3000 and PEG 4000 from 60 °C or 65 °C, respectively, up to 140 °C. As can be seen, the viscosity of PEG 4000 was higher than the viscosity of PEG 3000 at any given temperature. PEG 3000 and PEG 4000 can be categorized as Newtonian liquids [21] upon melting, as their viscosities were independent of the applied shear rate (Figure S3).

As dissolved CCX will also influence the viscosity of the polymer (in the compacts), the viscograms for PEG 3000 and PEG 4000 containing the maximum amount of dissolved CCX (i.e., 30 wt% CCX) are shown in Figure 1b. As CCX reduced the mobility of the PEG molecules, the viscosity was increased in the CCX–PEG mixtures as compared to the pure PEG systems at a target temperature.

To investigate the effect of viscosity at a target temperature, the compacts were exposed to microwave radiation at a target temperature of 76 °C, which in both cases is above the *T*_m_ of PEG 3000 and PEG 4000, and represents a temperature where the PEGs displayed significant differences in their viscosity, both in the presence and absence of dissolved CCX (Figure 1a,b). The temperature was set on the microwave oven, which continuously measured the temperature of the vials containing the compacts and adjusted the power output accordingly to reach and keep the set temperatures.

**Figure 2:**
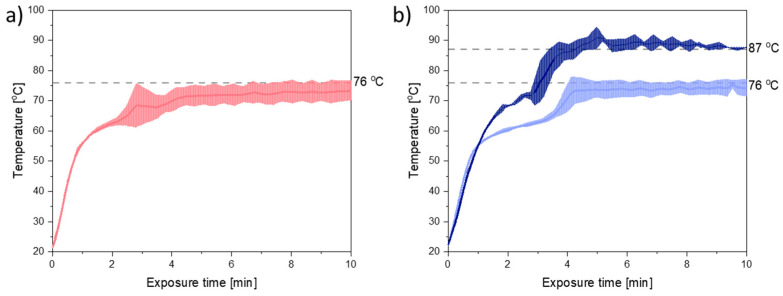
Temperature plots obtained during exposure to microwave radiation for (**a**) PEG 3000 compacts during 10 min of exposure at the target temperature of 76 °C (light red); (**b**) PEG 4000 compacts during 10 min of exposure at the target temperature of 76 °C (light blue) and 87 °C (dark blue). Mean (interpolated) ±SD (*n* = 3).

Figure 2 shows the temperature plots obtained during 10 min of exposure to microwave radiation at a target temperature of 76 °C. From Figure 2 it can be seen that the melting point of the mixtures of PEG and CCX (41–44 °C) was reached within the first minute of exposure to microwave radiation. Furthermore, the target temperature was nearly reached within 4–5 min for both compact containing PEG 3000 and PEG 4000.

During exposure to microwave radiation, the temperature, viscosity, and therefore also the diffusion coefficient of CCX in the melt, will change dynamically until the target temperature is reached and all CCX is dissolved. Hence, the rate of dissolution/amorphization of CCX into the polymer is expected to change during the initial 4–5 min of microwave radiation. However, as (i) the temperature profiles for PEG 3000 and PEG 4000 (Figure 2a,b (light blue)), did not vary significantly (*p* > 0.05) from each other and (ii) the viscosity of PEG 4000 was higher than the viscosity of PEG 3000, at all temperatures, with and without CCX (Figure 1a,b), the effect of the viscosity on the rate of amorphization of CCX could be evaluated with the present experimental setup.

To estimate the rate of amorphization, compacts prepared from both PEG 3000 and PEG 4000 were exposed to microwave radiation at a target temperature of 76 °C for 2, 4, 6, 8, or 10 min, and analyzed for crystallinity (i.e., presence of Bragg peaks) directly after exposure to microwave radiation using X-ray powder diffraction (XRPD).

**Figure 3:**
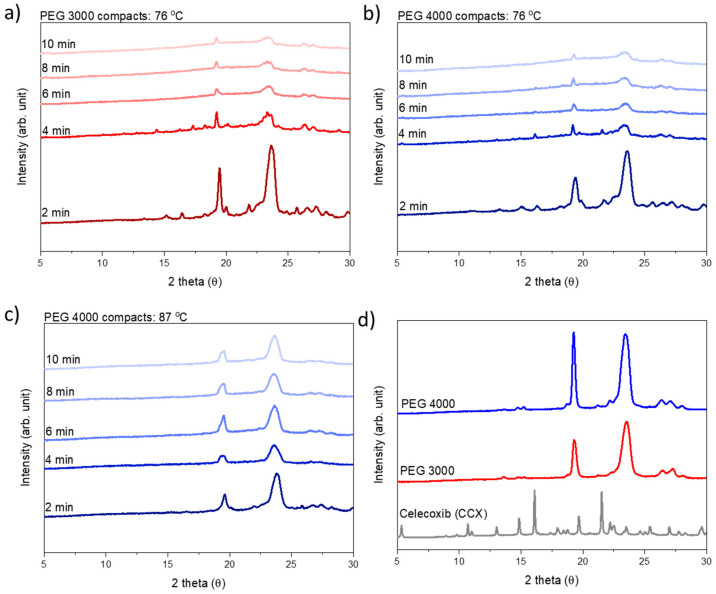
XRPD diffractograms for compacts exposed to microwave radiation for different times. (**a**) PEG 3000 at the target temperature of 76 °C; (**b**) PEG 4000 at the target temperature of 76 °C; (**c**) PEG 4000 at a target temperature of 87 °C and (**d**) pure substances for comparison. PEG 3000 compacts are colored red and PEG 4000 compacts are colored blue.

Following XRPD analysis, the compacts were categorized as amorphous either when a halo was obtained or the remaining diffraction peaks could be solely assigned to PEG, i.e., no crystalline CCX peaks were visible in the diffractograms (Figure 3). At a target temperature of 76 °C, PEG 3000 compacts required 6 min of exposure to microwave radiation to become fully amorphous, whilst PEG 4000 compacts required 10 min. As hypothesized, this can be explained by the viscosity of PEG 4000 compacts being higher as compared to PEG 3000, at all temperatures measured during microwave exposure (Figure 1 and Figure 2), and hence a faster rate of amorphization was obtained for CCX in the PEG 3000 compacts.

### 2.2. The Influence of Temperature on In Situ Amorphization of CCX in PEG (Target Viscosity)

As discussed in Section 2.1., the in situ amorphization of CCX in PEG is a dynamic process as both the temperature and the viscosity of the system will change during the exposure to microwave radiation, i.e., as a function of exposure time (Figure 1 and Figure 2). To evaluate the effect of temperature at a target viscosity, additional studies were conducted with 30 wt% CCX in PEG 4000 exposed to microwave radiation at a target temperature of 87 °C to obtain the same viscosity of the 30 wt% CCX in PEG 3000 at 76 °C.

From Figure 1b, it can be seen that in the temperature range of interest, i.e., 70–90 °C (note: due to practical limitations the temperature range of the microwave oven is reduced to 60–90 °C; due to practical limitations of the rheometer measurement the viscosity for PEG 4000 with 30 wt% CCX could only be measured from 70 °C onwards), a temperature difference of approx. 11 °C was needed to obtain the same viscosity for PEG 3000 and PEG 4000 with 30 wt% CCX. Hence, the target temperature was increased to 87 °C for PEG 4000 compacts. With these settings, the observed difference in the temperature profiles for PEG 3000 compacts exposed to 76 °C and PEG 4000 compacts exposed to 87 °C was approx. 11 °C (after the initial heating until 1.5 min) (Figure 2b), which means that the viscosity of the compacts was similar (Figure 2), and the effect of temperature could be evaluated. At the target temperature of 87 °C, compacts containing 30 wt% CCX and PEG 4000 became fully amorphous during exposure to microwave radiation after 4 min (Figure 3), which was faster than the 6 min necessary to obtain a full amorphization for the PEG 3000 compacts at a target temperature of 76 °C. In both cases, the systems had the same target viscosity, however, due to the higher temperature applied in the PEG 4000 compacts, the diffusion coefficient of CCX in the molten PEGs was increased resulting in faster rate of CCX amorphization in PEG 4000 compacts compared to PEG 3000 compacts. Hereby, the theory of a dissolution process of the drug into the polymer above the *T*_g_ of the polymer upon exposure to microwave radiation is further strengthened.

## 3. Materials and Methods

### 3.1. Materials

Celecoxib (CCX, *M*_w_ = 381.37 g/mol) and magnesium stearate (lubricant, *M*_w_ = 591.27 g/mol) were purchased from Fagron Nordic A/S (Copenhagen, Denmark). Polyethylene glycol 3000 (PEG 3000, *M*_w_ ≈ 3000 g/mol) and polyethylene glycol 4000 (PEG 4000, *M*_w_ ≈ 4000 g/mol) were kindly gifted by Merck (Darmstadt, Germany). All chemicals were used as received.

### 3.2. Rheological Measurements

The Discovery Hybrid Rheometer 3 (DHR 3) from TA Instruments Inc. (New Castle, DE, USA) with a cone plate geometry (ø of 40 mm, 1° angle) was used for the rheological measurements of PEG 3000, PEG 4000, as well as the mixtures of 30 wt% CCX and PEG 3000, and 30 wt% CCX and PEG 4000. The rheometer was equipped with a Peltier plate for temperature control and bearing, and gap size calibration was conducted before every measurement.

Temperature-dependent viscograms were obtained by placing the substances on the Peltier plate, which was set to 60 °C for PEG 3000 and 65 °C for PEG 4000 to obtain a melt. Subsequently, the gap between the cone geometry and the Peltier plate was reduced to the trim gap at 75 µm. Excess PEG was removed, and the gap was further reduced to the geometry gap of 25 µm. Subsequently, the samples were conditioned at the starting temperatures for 120 s before a shear rate of 100 s^−1^ was applied and the samples were heated to 140 °C at a rate of 5 °C/min.

Temperature-dependent viscograms for mixtures containing 30 wt% CCX and PEG (3000 or 4000) were obtained using the same method as described above. However, the initial temperature to obtain a melt was set to 65 °C for 30 wt% CCX PEG 3000 and to 70 °C for 30 wt% CCX PEG 4000. All experiments were conducted as a triplicate (*n* = 3).

Shear rate-dependent viscograms were obtained by conditioning PEG 3000 and PEG 4000 at 70 °C for 120 s followed by applying a shear rate from 5 s^−1^ to 200 s^−1^ within 300 s. The results were analyzed using the TRIOS software (version 4.1.1.) from TA Instruments Inc. (New Castle, DE, USA).

### 3.3. Compact Preparation

Physical powder mixtures containing 30 wt% celecoxib, 69 wt% PEG 3000 or PEG 4000 and 1 wt% magnesium stearate were prepared using mortar and pestle. Flat-faced 6 mm compacts containing approximately 100 ± 2 mg of the physical mixtures were prepared using an instrumented single punch tablet press GTP-1 from Gamlen Instruments (Nottingham, UK), which was fitted with a 500 kg load cell (CT6-500-022) at a compression pressure of 35 MPa. The compacts were stored over dried silica until further use.

### 3.4. In Situ Amorphization

In situ amorphization was induced by microwave radiation. For this, an Anton Paar Synthos 3000 microwave oven from Anton Paar GmbH (Graz, Austria) was used. The Anton Paar Synthos 3000 microwave oven was equipped with two magnetrons radiating unpulsed microwaves at a frequency of 2.45 GHz. The microwave oven was equipped with a 64MG5 rotor allowing for 3 rpm, giving space for 64 vials, from which 16 are temperature-recorded. The temperature of the vials was measured and recorded by a built-in IR probe in situ every 20 s. As the temperature was constantly measured, it could be set to a maximum, whilst the power was continuously adjusted to keep the maximum temperature constant. The maximal power output was set to 1000 W. Three compacts at a time were placed in individual sealed vials in temperature-recording positions. The remaining 13 temperature-recording positions in the rotor were equipped with empty vials to allow for accurate temperature measurements. Additionally, 16 sealed vials with each 1.5 mL demineralized water were placed in the rotor to absorb residual microwave radiation in non-temperature-recording positions. The sealed vials containing water had an offset to the vials containing compacts to avoid passive heating by conduction.

The compacts were exposed to microwave radiation until full amorphization was confirmed for 2, 4, 6, 8 and max. 10 min. PEG 3000 compacts were exposed to microwave radiation at a set temperature of 76 °C, and PEG 4000 compacts at 76 °C and 98 °C to achieve a target temperature of 76 °C and 87 °C, respectively. It should be noted that with the current experimental setup, the achievable temperature was limited to 90 °C (experimentally determined). By setting a higher temperature on the microwave oven, the heating profile could be adjusted, even though the temperature was not reached.

Each experiment was conducted as a triplicate in one microwave run (*n* = 3). The temperature data were corrected by the technical factor of 1.214 according to manufacturer guidelines.

### 3.5. Solid State Characterization

The solid state characteristics of the pure substances and the compacts exposed to microwave radiation were analyzed using X-Ray powder diffraction (XRPD). The compacts were measured by XRPD immediately after exposure to avoid recrystallization. The analysis was performed with a X’Pert Pro diffractometer from PANalytical (Eindhoven, The Netherlands) using Cu Kα radiation (λ = 1.54187 Å). The diffractograms were recorded from 5 to 30° 2θ at 45 kV and 40 mA. The substances or compacts were gently flattened on an aluminum plate. The diffractograms were analyzed with the X’Pert Data Viewer software (version 1.2.) from PANalytial (Eindhoven, The Netherlands).

### 3.6. Thermal Analysis

Thermal analysis was conducted using a Discovery differential scanning calorimeter (DSC) from TA Instruments (New Castle, DE, USA). All experiments were conducted under 50 mL/min nitrogen gas purge using Tzero aluminum pans with hermetically sealing lids with 3–5 mg powder. For the pure substances, PEG 3000 and PEG 4000, the onset of melting (*T*_m_) was determined by heating from 20 °C to 80 °C at a heating rate of 10 °C/min. Compact mixtures (see Section 3.3.) were additionally analyzed for onset of melting as well as their monotectic behavior. The mixtures were heated at a ramp of 2 °C/min from 0 °C to 180 °C.

The thermal specifications were analyzed using the TRIOS software (version 4.1.1) from TA Instruments Inc. (New Castle, DE, USA).

## 4. Conclusions

In this study, it was shown that the rate of in situ amorphization of CCX in PEG 3000 and PEG 4000 was dependent on both the viscosity and the temperature of the molten PEGs. Upon exposure to microwave radiation at a higher temperature, a lower viscosity was obtained, which resulted in a higher diffusion coefficient of the drug CCX in the mobile polymers. A higher diffusion coefficient increased the rate of amorphization, which further strengthens the hypothesis of an underlying dissolution process of the drug in a mobile polymer upon exposure to microwave radiation.

## Data Availability

Data available upon request.

## Supplementary Materials

The following are available online, Figure S1: DSC thermograms of melting points of PEG 3000 and PEG 4000; Figure S2: DSC thermograms of 30 wt % CCX in PEG 3000 and PEG 4000 showing monotectic behavior; Figure S3: Viscograms for PEG 3000 and PEG 4000.
