# Supplementary material for: The Influence of Temperature and Viscosity of Polyethylene Glycol on the Rate of Microwave-Induced In Situ Amorphization of Celecoxib
Source: Molecules. 2020 Dec 29;26(1):110. doi: 10.3390/molecules26010110 (PMC7796040; doi:10.3390/molecules26010110)
Supplement: Supplementary file 1 [file molecules-26-00110-s001.pdf]

Supplementary material:

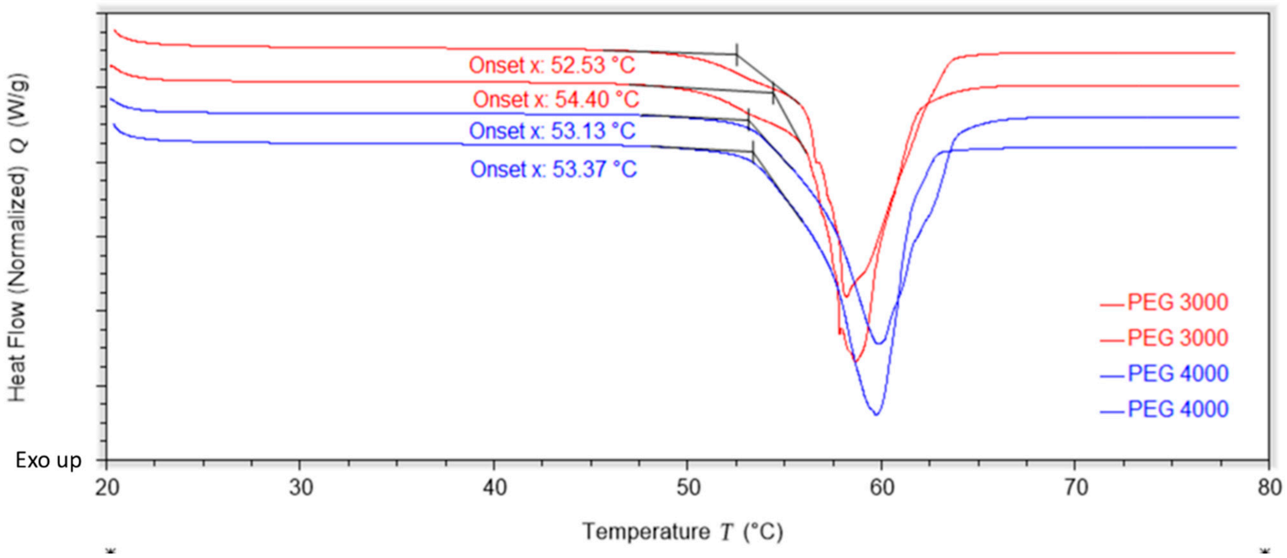

Figure S1: Melting points of PEG 3000 (red) and PEG 4000 (blue). Exo up. No y-scaling due to overlay (*n* = 2).

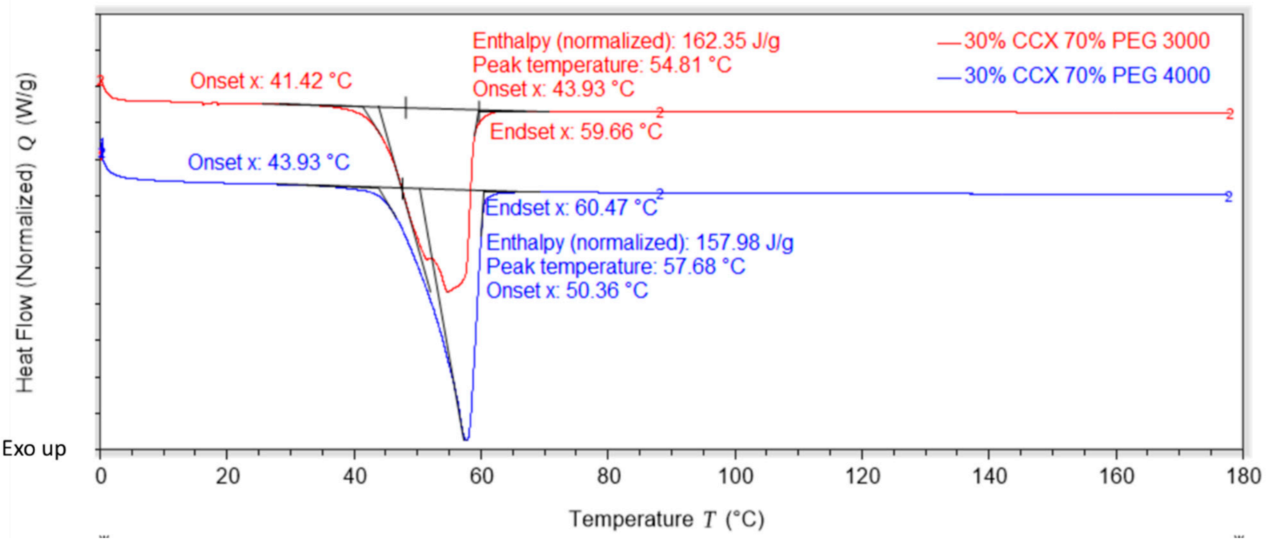

Figure S2: Monotecticum formation of 30% CCX with 70% PEG 3000 (red) and PEG 4000 (blue). Exo up. No y-scaling due to overlay. (Note: different kinetics and heating mechanism in the DSC and the microwave oven).

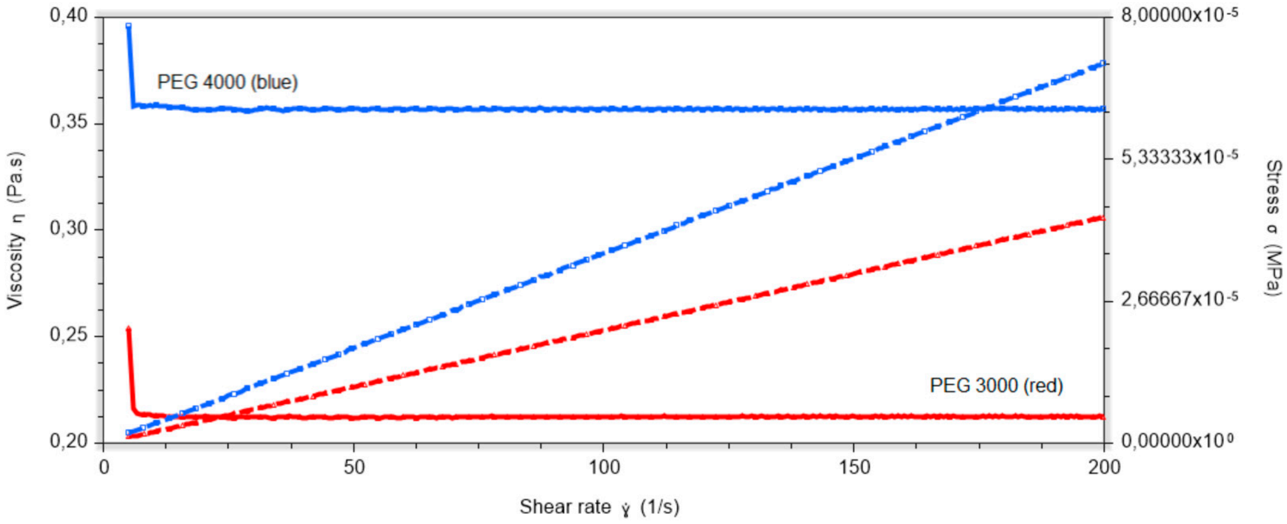

Figure S3: Shear-rate dependent viscograms obtained for PEG 3000 (red) and PEG 4000 (blue). The solid lines show the viscosity as a function of the shear rate (left y-axis). The dashed lines show the stress as a function of the shear rate (right y-axis) (*n* = 1).
